# Predicting 3-month prognosis of cerebral venous thrombosis: a machine learning approach incorporating inflammatory markers

**DOI:** 10.3389/fneur.2026.1843121

**Published:** 2026-08-11

**Authors:** Huanxiang Huang, Fan Chen, Ying Chen, Shaoyong Lin, Xinhua Tian, Shuwen Mu, Jun Li, Pengwei Hou, Shuling Chen, Chunfa Wu, Liangfeng Wei, Shousen Wang, Ziqi Li

**Affiliations:** 1 Department of Neurosurgery, Fuzong Clinical Medical College of Fujian Medical University, Fuzhou, Fujian, China; 2 Department of Neurosurgery, Dongfang Affiliated Hospital of Xiamen University, School of Medicine, Xiamen University, Xiamen, Fujian, China; 3 Fujian Provincial Clinical Medical Research Center for Minimally Invasive Diagnosis and Treatment of Neurovascular Diseases, Fuzhou, Fujian, China; 4 Department of Science and Education, Fuzong Clinical Medical College of Fujian Medical University, Fuzhou, Fujian, China; 5 Department of Neurosurgery, The Affiliated Zhongshan Hospital of Xiamen University, Xiamen, Fujian, China; 6 Aberdeen Medical School, College of Life Sciences and Medicine, University of Aberdeen, Aberdeen, United Kingdom; 7 Department of Neurosurgery, Jinjiang Municipal Hospital (Shanghai Sixth People's Hospital), Jinjiang, Fujian, China; 8 Department of Neurosurgery, The First Hospital of Shanxi Medical University, Taiyuan, Shanxi, China

**Keywords:** cerebral venous thrombosis, inflammatory marker, machine learning, prognosis, support vector machine

## Abstract

This study aimed to explore a machine learning (ML) model for predicting the long-term prognosis of Cerebral Venous Thrombosis (CVT). We conducted a comprehensive retrospective analysis on CVT patients admitted to Fujian Medical University Fuzhou General Hospital and The Affiliated Zhongshan Hospital of Xiamen University from January 1, 2020 to December 31, 2023. The retrospective cohort of 350 patients was used for model development using a 5-fold nested cross-validation framework. To address class imbalance, the Borderline-SMOTE technique was applied within the internal training folds. A total of 31 clinical and laboratory variables were evaluated as potential predictors. Lasso and Elastic Net (cv-Enet) were employed for preliminary variable selection, and the intersection of these two methods yielded 11 key variables. Random Forest was then applied to further reduce dimensionality, resulting in a final set of 5 crucial variables. These variables were ranked by importance using SHapley Additive exPlanation (SHAP) analysis. In internal validation using nested cross-validation, the SVM model achieved an area under the receiver operating characteristic curve (AUROC) of 0.870, with a sensitivity of 0.784 and a specificity of 0.897. This performance was corroborated in an independent, prospectively collected external cohort of 152 patients from the First Hospital of Shanxi Medical University (January 1, 2023 to December 31, 2025) where the model demonstrated robust discrimination with an AUROC of 0.862 (95% CI: 0.829–0.884). However, calibration analysis indicated a slight overestimation of risk (calibration-in-the-large ≈ 9%), suggesting that the model currently serves best as a risk stratification adjunct rather than a definitive outcome predictor. Bootstrap resampling confirmed the stability of the discriminative performance. Exploratory subgroup and fairness analyses suggested stable model performance across key demographic subgroups. Our study focused on the development and validation of an ML-based model for long-term CVT prognosis prediction, highlighting the significant role of inflammatory markers. These findings indicate that the SVM model, with further calibration and prospective validation, could serve as a useful adjunct for risk stratification in CVT.

## Introduction

Cerebral Venous Thrombosis (CVT) is a distinct and clinically significant subtype of cerebrovascular disease, accounting for approximately 0.5–1% of all stroke cases (1). This condition predominantly affects young and middle-aged populations, with a particular inclination towards women. The incidence rate in Western countries is estimated to be between 1.3 and 1.6 per 100,000 individuals, and emerging data suggest even higher rates in Asia and the Middle East (2, 3). Unlike arterial strokes, CVT primarily involves the dural sinuses or cortical veins, resulting in a diverse array of clinical manifestations that can encompass headaches, seizures, and focal neurological deficits.

The etiology of CVT is multifaceted, involving a myriad of risk factors that include both inflammatory and non-inflammatory elements, such as infections, autoimmune conditions, hypercoagulable states, and vascular injuries (4). Despite advancements in diagnostic modalities, particularly Magnetic Resonance Imaging (MRI) and Magnetic Resonance Venography (MRV) (5), the long-term prognosis of CVT remains a subject of clinical concern. Approximately 13% of CVT patients either succumb to the condition or remain handicapped, as indicated by a modified Rankin Scale (mRS) score greater than 2 (i.e., ≥3) (6). Some patients may develop severe clinical manifestations like cerebral venous infarction/hemorrhage, seizures, mental status impairment, and disturbance of consciousness, as measured by a Glasgow scale score of less than 9 (7–10). These severe outcomes accentuate the clinical urgency and value of research focused on the prognosis of CVT.

In recent years, the role of inflammation in CVT has garnered increasing attention. Inflammation serves as both a predisposing and exacerbating factor in CVT, contributing to localized hypercoagulation and endothelial cell injury (4, 11). Infections and autoimmune diseases can trigger inflammatory responses, leading to hypercoagulable states and endothelial dysfunction, which are high-risk factors for CVT (12). Inflammatory mediators, including cytokines and chemokines, play a crucial role in the pathogenesis of CVT (13), exacerbating cerebral ischemia and leading to more severe clinical manifestations (14). In terms of inflammatory markers, a range of indicators have been identified as relevant to disease prognosis (15, 16). These include not only C-reactive protein (CRP) and white blood cell counts, but also other markers such as the Platelet to Lymphocyte Ratio (PLR), Neutrophil to Lymphocyte Ratio (NLR), Lymphocyte to Monocyte Ratio (LMR), the Glasgow Prognostic Score (GPS), Systemic immune-inflammation index (SII), C-reactive protein to albumin ratio (CRP/Alb ratio), and Neutrophil–Platelet Score (NPS). These markers not only aid in diagnosis but are also useful for assessing treatment response and predicting long-term outcomes. Despite these insights, there remains a notable absence of reliable predictive models for the long-term prognosis of CVT that incorporate these inflammatory markers.

Machine Learning (ML) has garnered considerable attention in healthcare for its remarkable predictive capabilities. These algorithms are increasingly being used to optimize clinical diagnoses and treatment regimens, demonstrating substantial potential for improving patient outcomes (17–19). Among the various ML algorithms, the Support Vector Machine (SVM) stands out for its exceptional performance. SVM is particularly advantageous because it offers high predictive accuracy while requiring relatively low computational power, making it an ideal choice for real-time clinical applications. Its mathematical foundation, based on hyperplane separation in a high-dimensional space, allows for effective classification and regression tasks, which are critical in medical settings for disease identification and prognosis prediction (20, 21).

However, the intricate nature of ML models, including SVM, often necessitates a level of interpretability to make the outcomes clinically actionable. This is where SHapley Additive exPlanation (SHAP) analysis comes into play (22). Increasingly employed in medical research, SHAP provides localized, intuitive explanations for model predictions, thereby enhancing both the credibility and the clinical applicability of the model. By integrating SHAP with SVM, healthcare professionals can gain not only accurate but also interpretable insights, facilitating more informed and reliable clinical decisions.

The main contributions of this study were as follows: (1) We introduced an effective approach that innovatively applied machine learning techniques to develop and validate a model for predicting the long-term prognosis of CVT. (2) This multicenter study first combines prospective and retrospective methodologies to investigate the role of inflammation in cerebral venous thrombosis (CVT). To our knowledge, this is the first study to incorporate the Glasgow Prognostic Score (GPS) and Neutrophil–Platelet Score (NPS) into a prognostic model for CVT, representing the largest two-cohort multicenter analysis of inflammatory markers in CVT to date. (3) We offered localized, visual explanations of inflammatory markers and their direct outcomes, enhancing the model’s interpretability. Additionally, we conducted a fairness analysis to ensure the model’s applicability across diverse clinical scenarios. This comprehensive approach aimed to provide a robust, interpretable, and clinically useful tool for better understanding and managing CVT.

## Methods

For the retrospective development cohort (January 1, 2020 to December 31, 2023), the requirement for informed consent was waived by the corresponding ethics committees because of the retrospective design and the use of anonymized clinical data. For the prospective external validation cohort (January 1, 2023 to December 31, 2025), written informed consent was obtained from all participants or their legal proxies prior to enrollment. All patient data were anonymized prior to analysis, and the authors had no access to information that could identify individual participants.

### Participants

A total of 350 consecutive patients diagnosed with CVT were retrospectively enrolled from two tertiary hospitals, including Fuzong Hospital of Fujian Medical University and the Affiliated Zhongshan Hospital of Xiamen University, between January 1, 2020 and December 31, 2023. This cohort constituted the development dataset.

In addition, an independent prospective external validation cohort of 152 CVT patients was collected from the First Hospital of Shanxi Medical University between January 1, 2023 and December 31, 2025. Patients were excluded if they had acute ischemic stroke, acute myocardial infarction, renal or hepatic failure, malignancy, deep vein thrombosis, pulmonary embolism, definite autoimmune disease, acute or chronic infection, or use of anti-inflammatory medication within four weeks prior to blood sampling, or if they refused blood sampling or lumbar puncture.

Following Riley et al. (23) guidance for prediction model development, we estimated the required sample size. With 31 candidate predictors and an expected outcome proportion of 0.32, the events-per-variable (EPV) was 112/31 ≈ 3.6, which is below the commonly recommended minimum of 10–20. Using Riley’s formula to achieve a target shrinkage factor of ≥0.9, the required sample size was estimated to be approximately 480 patients. Our development cohort of 350 patients is therefore modest and may limit model complexity. To mitigate the risk of overfitting, we employed L1/L2 regularization (LASSO and Elastic Net), performed strict feature selection within nested cross-validation, and retained a parsimonious final model with only five predictors. The independent external validation further confirmed acceptable generalizability, though the limited sample size remains a notable limitation that should be addressed in future larger-scale studies.

#### Variables

The following 31 variables were included: baseline blood biomarkers (including NPS, GPS, CRP, LMR, NLR, and PLR) and demographic and clinical variables (including sex, age, height, weight, smoking history, and history of hypertension) (Table 1). Categorical variables were transformed into binary indicators using one-hot encoding.

**Table 1:** Demographics and clinical characteristics of the study population: retrospective and prospective cohorts.

Variable	All (*n* = 502)	Retrospective (*n* = 350)	Prospective (*n* = 152)	*P* value*
Demographics				
Age, years, median [IQR]	41.0 [34.5, 48.3]	41.2 [34.8, 48.7]	40.7 [33.9, 47.5]	0.559
Female, *n* (%)	194 (38.65)	133 (38.00)	61 (40.13)	0.731
Male, *n* (%)	308 (61.35)	217 (62.00)	91 (59.87)	
Comorbidities				
Hypertension, *n* (%)	110 (21.91)	72 (20.57)	38 (25.00)	0.571
Diabetes, *n* (%)	59 (11.75)	38 (10.86)	21 (13.82)	0.457
Smoking, *n* (%)	44 (8.76)	29 (8.29)	15 (9.89)	0.398
Clinical presentation				
Headache, *n* (%)	389 (77.49)	273 (78.00)	116 (76.32)	0.501
Consciousness dysfunction, *n* (%)	58 (11.55)	45 (12.86)	13 (8.55)	0.061
Heart rate, bpm, median [IQR]	78 [71, 87]	77 [70, 85]	80 [72, 89]	0.682
Respiratory rate, /min, median [IQR]	18 [16, 20]	18 [16, 20]	17 [16, 19]	0.608
Glasgow prognostic score (GPS)				<0.001
0, *n* (%)	131 (26.10)	77 (22.00)	54 (35.53)	
1, *n* (%)	247 (49.20)	186 (53.14)	61 (40.13)	
2, *n* (%)	124 (24.70)	87 (24.86)	37 (24.34)	
Therapy				
Anticoagulation therapy, *n* (%)	296 (58.96)	205 (58.57)	91 (59.87)	0.785
Thrombectomy, *n* (%)	93 (18.53)	65 (18.57)	28 (18.42)	0.968
Decompressive craniectomy, *n* (%)	41 (8.17)	29 (8.29)	12 (7.89)	0.875
Laboratory biomarkers				
White blood cell (×10^9^/L), median [IQR]	7.60 [6.80, 8.60]	7.70 [6.80, 8.60]	7.50 [6.70, 8.50]	0.595
Hemoglobin (g/L), median [IQR]	129.0 [120.2, 137.8]	129.5 [120.8, 138.2]	128.3 [119.6, 137.5]	0.045
C-reactive protein (mg/L), median [IQR]	12.52 [9.13, 16.95]	13.86 [10.25, 19.00]	10.05 [8.07, 14.33]	<0.001
Platelet (×10^9^/L), median [IQR]	277.4 [230.0, 325.0]	279.5 [235.0, 330.0]	272.5 [220.0, 320.0]	0.004
Thrombin time (s), median [IQR]	11.6 [11.5, 11.7]	11.7 [11.6, 11.8]	11.5 [11.3, 11.7]	0.013
Fibrinogen (g/L), median [IQR]	3.82 [3.32, 4.32]	3.81 [3.34, 4.29]	3.85 [3.28, 4.37]	0.512
Prothrombin time (s), mean ± SD	12.91 ± 1.22	12.80 ± 1.15	13.10 ± 1.31	0.091
Lactate (mmol/L), mean ± SD	4.78 ± 1.05	4.68 ± 0.92	4.91 ± 1.28	0.012
Lactate dehydrogenase (U/L), median [IQR]	89.6 [73.3, 110.9]	91.3 [75.3, 110.9]	88.8 [73.0, 105.6]	0.038
AST (U/L), median [IQR]	33.1 [25.2, 44.6]	31.8 [24.6, 41.3]	35.2 [26.2, 47.8]	0.044
ALT (U/L), median [IQR]	27.7 [20.8, 37.1]	27.2 [20.7, 36.6]	28.5 [21.6, 38.7]	0.221
LMR, median [IQR]	2.81 [2.20, 3.43]	2.52 [2.04, 3.22]	2.96 [2.25, 3.66]	0.018
NLR, median [IQR]	3.54 [2.60, 4.31]	3.75 [2.80, 5.00]	3.05 [2.41, 3.59]	0.014
PLR, median [IQR]	154.2 [125.0, 188.0]	153.5 [125.0, 186.0]	157.0 [122.0, 200.0]	<0.001
SII, median [IQR]	887.6 [710.1, 1259.0]	871.2 [605.3, 1298.6]	910.8 [731.6, 1216.8]	0.017

*P values compare retrospective (*n* = 350) and prospective (*n* = 152) cohorts.

#### Outcomes

The functional prognosis at 3 months after discharge, assessed using the modified Rankin Scale (mRS) (24), served as the primary outcome. The 3-month mRS was ascertained via telephone follow-up and was dichotomized into good outcome (mRS ≤ 1) versus poor outcome (mRS > 1). mRS scores at admission and discharge were documented for descriptive purposes. Follow-up completeness was high, with a loss-to-follow-up rate of less than 5%. Patients with missing 3-month mRS assessments were excluded from outcome analyses. To ensure comparability with prior CVT literature, we performed a sensitivity analysis redefining poor outcome as mRS ≥ 3 (i.e., mRS ≤ 2 as good outcome). In this sensitivity analysis, the SVM model achieved an AUROC of 0.865 (95% CI: 0.83–0.90) in internal validation and 0.850 (95% CI, 0.81–0.89) in external validation, confirming that the model’s discriminative performance was robust to the choice of dichotomization threshold. The primary analysis using the stricter mRS ≤ 1 versus >1 definition was retained to identify patients with complete or near-complete functional independence. The 3-month mRS was ascertained via telephone by trained research assistants who were blinded to all predictor variables. A structured questionnaire based on the validated mRS interview protocol was used for all follow-up assessments. Inter-rater reliability was assessed for a random subset of 30 patients by two independent raters, yielding a kappa coefficient of 0.89 (95% CI, 0.82–0.95), indicating excellent agreement.

### Data pre-processing and feature selection

During data preprocessing, binary missing-indicator variables were created to denote the presence of missing values. Missing data were assumed to be missing at random based on clinical review and were imputed using the mean for continuous variables and the mode for categorical variables. To evaluate the robustness of the imputation strategy, a sensitivity analysis using multiple imputation was additionally performed. Five imputed datasets were generated, with the same candidate predictors and outcome variable included in the imputation model, and the full modeling pipeline was repeated after imputation. Over-sampling using the Borderline–Synthetic Minority Oversampling Technique (Borderline-SMOTE) was applied only within the inner-loop training folds of the nested cross-validation framework (25); no resampling was performed in the outer validation folds or in the external validation cohort, thereby minimizing the risk of information leakage.

Feature selection was performed within the nested cross-validation framework to minimize the risk of information leakage. For each outer-loop split, all preprocessing and feature selection procedures, including missing-value imputation, feature scaling, LASSO selection, Elastic Net selection, and subsequent Random Forest–based feature reduction, were fitted exclusively on the corresponding training data. The fitted preprocessing parameters and selected feature set were then applied to the held-out validation fold without refitting. This procedure was repeated independently across all outer folds. No information from the validation folds or the external validation cohort was used for imputation, scaling, feature selection, hyperparameter tuning, or model training. To enhance transparency, a schematic diagram and pseudocode of the nested feature selection pipeline are provided in the Supplementary material.

Predictors consistently selected by both LASSO and elastic net models were retained and further refined using a random forest (RF)–based importance ranking. Bootstrap resampling within the training data was applied to assess the stability of selected features. To enhance model interpretability, an eXtreme Gradient Boosting (XGBoost) model was fitted using the retained predictors, and SHapley Additive exPlanations (SHAP) values were calculated to quantify the relative contribution of each feature.

### Model building and evaluation

A support vector machine (SVM) classifier was used to construct the final predictive model based on the selected features. Hyperparameters were optimized within the inner loop of the nested cross-validation framework using five-fold cross-validation. The area under the curve (AUC), sensitivity, and specificity were used to quantify model performance during internal validation.

The final trained model was subsequently applied once to the independent prospective cohort for external validation. Model performance was assessed using receiver operating characteristic (ROC) and precision–recall (PR) curves, and corresponding performance metrics were calculated at the predefined operating threshold. Model calibration was evaluated using calibration curves to assess agreement between predicted and observed probabilities.

All performance metrics were calculated on the external cohort without any model refitting. To quantify uncertainty in external validation performance, bootstrap resampling (2,000 replications) was applied to the external validation cohort to estimate 95% confidence intervals for all performance metrics. Exploratory subgroup analyses were conducted to descriptively examine model discrimination across predefined age and sex strata; no formal hypothesis testing was performed given the limited sample size and the exploratory nature of these analyses. In addition, logistic regression models incorporating interaction terms were fitted to assess potential differential model performance across age, sex, and therapeutic intervention groups.

The support vector machine (SVM) classifier was constructed using a radial basis function (RBF) kernel to capture potential nonlinear relationships between predictors and outcomes. Hyperparameters were optimized within the inner loop of the nested cross-validation framework using grid search with five-fold cross-validation.

The search space included a log-spaced grid with C ∈ {0.01, 0.1, 1, 10, 100} and gamma ∈ {0.001, 0.01, 0.1, 1}. The optimal hyperparameters selected based on mean cross-validated AUROC were C = 1.0 and gamma = 0.1.

Model calibration was performed using isotonic regression. The calibration model was fitted using predicted probabilities derived from the training data and subsequently applied unchanged to the external validation cohort to avoid information leakage. Primary external validation was performed without any model refitting, recalibration, or threshold adjustment. In addition, an exploratory *post hoc* logistic recalibration analysis was conducted separately in the external cohort to assess whether probability estimation could be improved in a new clinical setting. No data from the external validation cohort were used for feature selection, hyperparameter tuning, calibration, or threshold determination.

After nested cross-validation, the final SVM model was retrained on the entire development cohort using the optimal hyperparameters identified in the inner-loop cross-validation.

### Statistical analysis

All data analyses were conducted using Python (version 3.9.16). The following libraries were used: pandas (1.5.3) for data manipulation and preprocessing; numpy (1.23.5) for numerical computation; scikit-learn (1.2.2) for feature selection, model development, hyperparameter tuning, and performance evaluation; imbalanced-learn (0.10.1) for Borderline-SMOTE implementation; xgboost (1.7.6) for feature importance analysis; shap (0.41.0) for model interpretability analysis; matplotlib (3.7.1) and seaborn (0.12.2) for data visualization; and statsmodels (0.13.5) for statistical analysis in subgroup evaluation. All stochastic processes were controlled using a fixed random seed (seed = 42) to ensure reproducibility.

Categorical variables were compared using the χ^2^ test or Fisher’s exact test, as appropriate. Continuous variables were compared using Student’s t-test, analysis of variance, or the Mann–Whitney U test, as appropriate. Confidence intervals for sensitivity, specificity, positive predictive value, negative predictive value, and accuracy were calculated using the Clopper–Pearson method. A two-sided *p* value < 0.05 was considered statistically significant. The relationship between the number of candidate predictors and outcome events was considered with reference to contemporary recommendations for clinical prediction model development (23). The detailed implementation of the nested cross-validation and feature selection pipeline is provided in Supplementary Methods S1. The analysis pipeline, including code for data preprocessing, feature selection, and model training, is openly available in the public repository mentioned above.

To benchmark the SVM model, we additionally fitted three alternative classifiers under the same nested cross-validation framework: penalized logistic regression (with L2 regularization), random forest (with 100 trees and default hyperparameters), and XGBoost (with default hyperparameters). Model performance was compared using mean AUROC and Brier score.

## Results

### Baseline characteristics

A total of 730 patients with suspected CVT were initially screened across the three participating centers. After applying the exclusion criteria, 350 patients were included in the development cohort and 152 in the external validation cohort. The numbers of patients excluded for each criterion were: acute ischemic stroke (*n* = 89), acute myocardial infarction (*n* = 34), renal or hepatic failure (*n* = 28), malignancy (*n* = 34), deep vein thrombosis or pulmonary embolism (*n* = 19), definite autoimmune disease (*n* = 23), acute or chronic infection (*n* = 45), anti-inflammatory medication use within four weeks (*n* = 41), and refusal of blood sampling or lumbar puncture (*n* = 22). Some patients met multiple exclusion criteria. A detailed flowchart is provided as Supplementary Figure S1. Although we excluded patients with overt inflammatory conditions to reduce confounding, we acknowledge that this may limit generalizability; however, the strong performance of inflammatory markers in the remaining cohort underscores their importance even in the absence of major systemic inflammation.

The study included 350 patients, among whom 112 (32.0%) experienced a poor outcome (mRS > 1) at 3 months. The entire retrospective cohort was used within a nested cross-validation framework for model development and internal validation; therefore, no fixed single hold-out test set was predefined. The missing rate of the collected data ranged from 0.00 to 4.00%. The proportion of missing data for each variable is detailed in Supplementary Table S1. Sensitivity analysis using multiple imputation (five imputations) yielded similar results, with an AUROC of 0.868 compared to 0.870 in the primary analysis, and no substantial change in the selected feature set.

To quantify the distributional shift between the development and external validation cohorts, we calculated the Kolmogorov–Smirnov (KS) distance for the five final predictors. The KS distances were: CRP = 0.32, PLR = 0.28, SII = 0.21, GPS = 0.35, and NPS = 0.18. The largest shifts were observed for CRP and GPS, which coincided with the calibration drift (calibration slope 1.142) observed in the external cohort. This suggests that differences in baseline inflammatory profiles across centers may partly explain the model’s tendency toward overconfident predictions, highlighting the need for site-specific calibration before clinical deployment.

### Distribution of outcomes across cohorts and centers

The detailed distribution of patients and outcomes across all contributing centers is presented in Table 2 (development cohort) and Table 3 (external validation cohort).

**Table 2:** Distribution of patients and outcomes in the development cohort by contributing center.

Center	Total patients (n)	Good outcome (mRS ≤ 1)	Poor outcome (mRS > 1)	Poor outcome rate
Center 1 (Fuzong Hospital)	200	140	60	30.0%
Center 2 (Zhongshan Hospital)	150	98	52	34.7%
Overall development cohort	350	238	112	32.0%

**Table 3:** Distribution of patients and outcomes in the independent external validation cohort.

Center	Total patients (n)	Good outcome (mRS ≤ 1)	Poor outcome (mRS > 1)	Poor outcome rate
Center 3 (First Hospital of Shanxi Medical University)	152	106	46	30.3%

**Figure 1:**
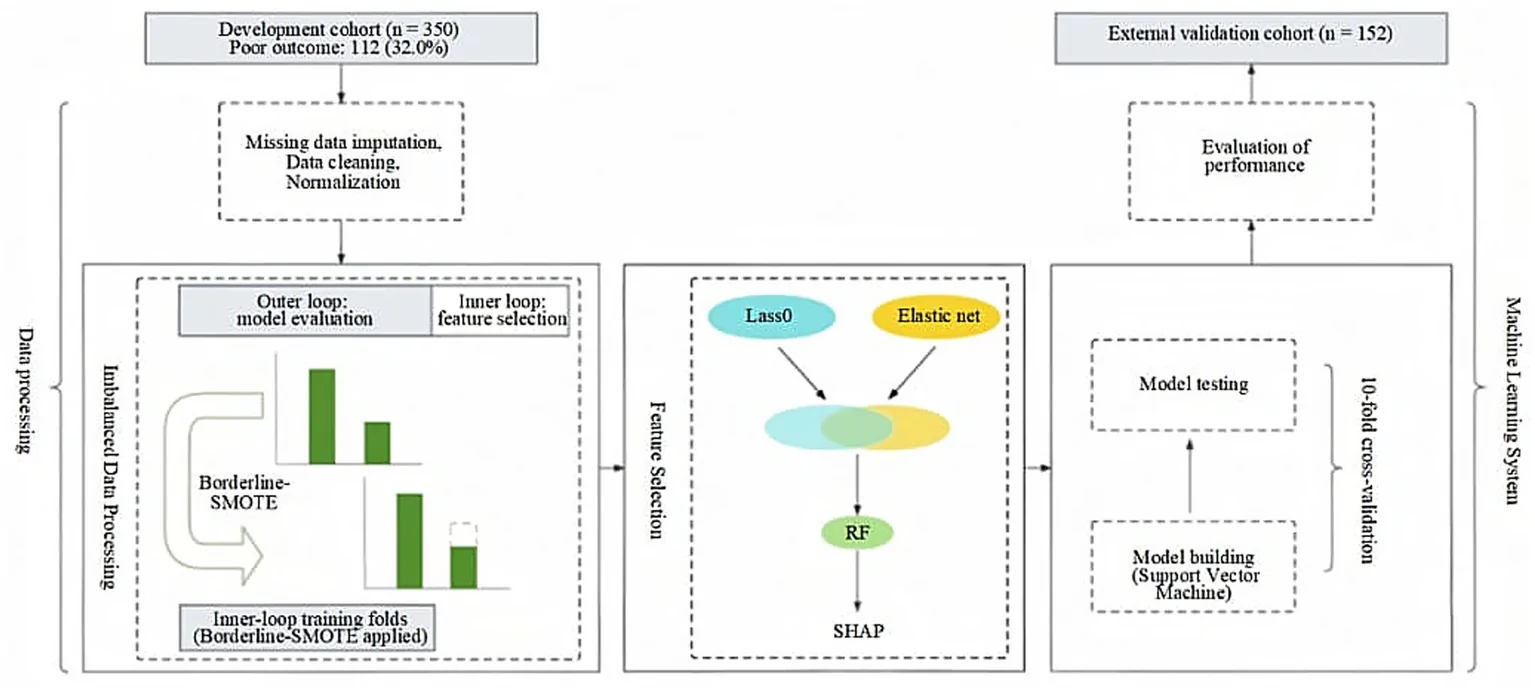
Workflow of the machine learning model development and validation. The development cohort (*n* = 350) underwent data preprocessing, including missing data imputation, data cleaning, and normalization. Model development was performed using nested five-fold cross-validation. Within each inner-loop training fold, class imbalance was addressed using the Borderline-Synthetic Minority Over-sampling Technique (Borderline-SMOTE), followed by feature selection and model construction using multiple machine learning approaches. Model performance was evaluated in the outer-loop validation folds. The finalized model was then applied once to an independent prospective external validation cohort (*n* = 152) from the First Affiliated Hospital of Shanxi Medical University.

The development cohort comprised a total of 350 patients retrospectively enrolled from 2 centers between January 1, 2020 and December 31, 2023, including Fuzong Hospital of Fujian Medical University, the Affiliated Zhongshan Hospital of Xiamen University. This cohort was used for model development and internal validation within a 5-fold nested cross-validation framework, and no fixed hold-out test set was predefined (Figure 1).

### Feature selection and importance

**Figure 2:**
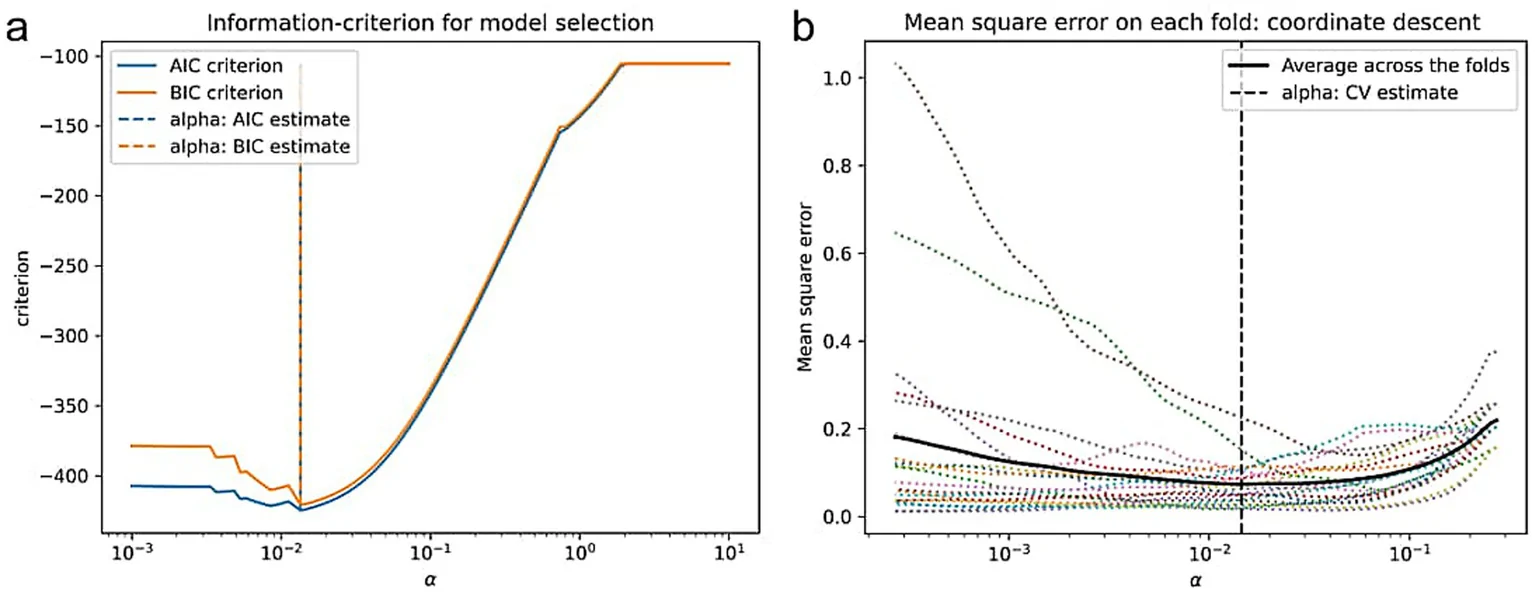
Feature selection and validation using LASSO analysis. **(a)** The blue and orange curves represent changes in the Akaike Information Criterion (AIC) and Bayesian Information Criterion (BIC) as functions of the regularization parameter *α*. The blue and orange dashed lines indicate the optimal *α* values based on the AIC and BIC criteria, respectively. **(b)** Each dotted curve represents one fold in cross-validation. The solid black curve indicates the mean squared error (MSE) averaged across folds, and the vertical dashed line marks the optimal *α* value determined by cross-validation.

**Figure 3:**
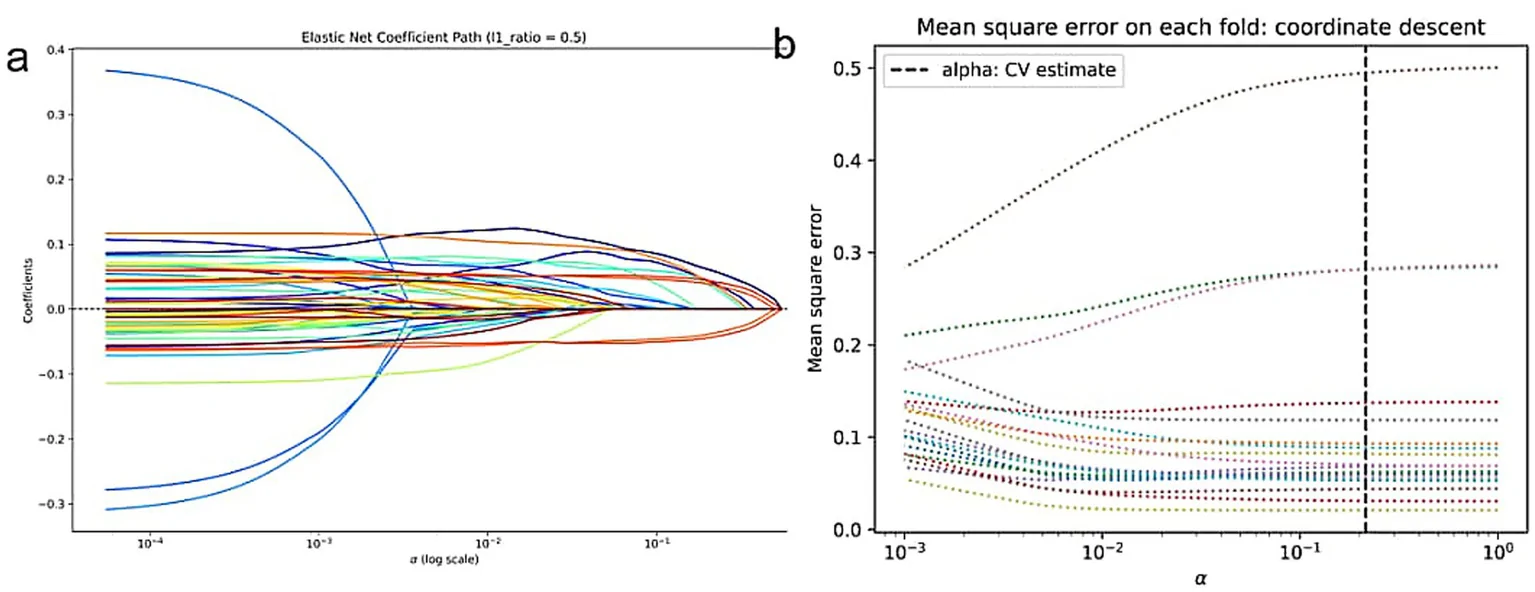
Feature selection and validation using Elastic Net (Enet) analysis. **(a)** Coefficient trajectories of candidate variables across different values of the regularization parameter *α*. **(b)** Each dotted curve represents one fold in cross-validation, and the vertical dashed line indicates the optimal *α* value estimated by cross-validation.

**Figure 4:**
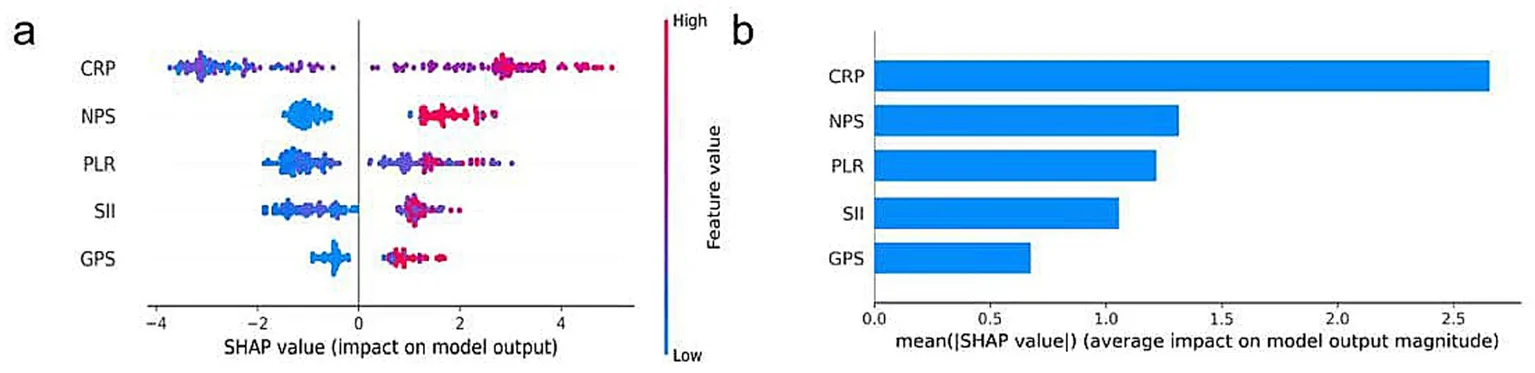
Feature importance based on SHAP analysis. **(a)** Distribution of SHA*p* values for the selected features in the final model. **(b)** Relative contribution and ranking of each feature according to their mean absolute SHAp values.

The feature selection process was embedded within each fold of the nested cross-validation. To illustrate the selection rationale, we first present the results from a single representative run on the entire development cohort. Lasso regression identified 18 candidate variables (Figure 2). Elastic Net regression identified 15 variables (Figure 3a). The intersection of these two sets yielded 11 variables. These 11 variables were subsequently refined using Random Forest, resulting in a final set of 5 variables (CRP, PLR, SII, NPS, GPS) that demonstrated high selection stability across bootstrap resampling. This set of variables was consistently identified as the most important across the majority of the nested cross-validation folds. Their relative contribution to the model’s predictions was further elucidated using SHAP analysis (Figures 4a,b).

We assessed multicollinearity among the five final predictors using variance inflation factors (VIFs). All VIFs were below 5 (CRP: 2.1, PLR: 3.4, SII: 4.2, NPS: 3.8, GPS: 2.9), indicating acceptable collinearity. The correlation matrix (Supplementary Table S2) showed moderate correlations between PLR and SII (*r* = 0.62) and between CRP and GPS (*r* = 0.55), but no coefficients exceeded 0.7, suggesting that while some redundancy exists, each variable retains a degree of independent information. We additionally computed SHAP interaction values to examine potential synergistic effects; the strongest interaction was observed between CRP and GPS, consistent with the structural overlap where GPS incorporates CRP in its calculation. Despite this partial redundancy, both markers retained independent marginal contributions to the model.

### Model development and evaluation

**Figure 5:**
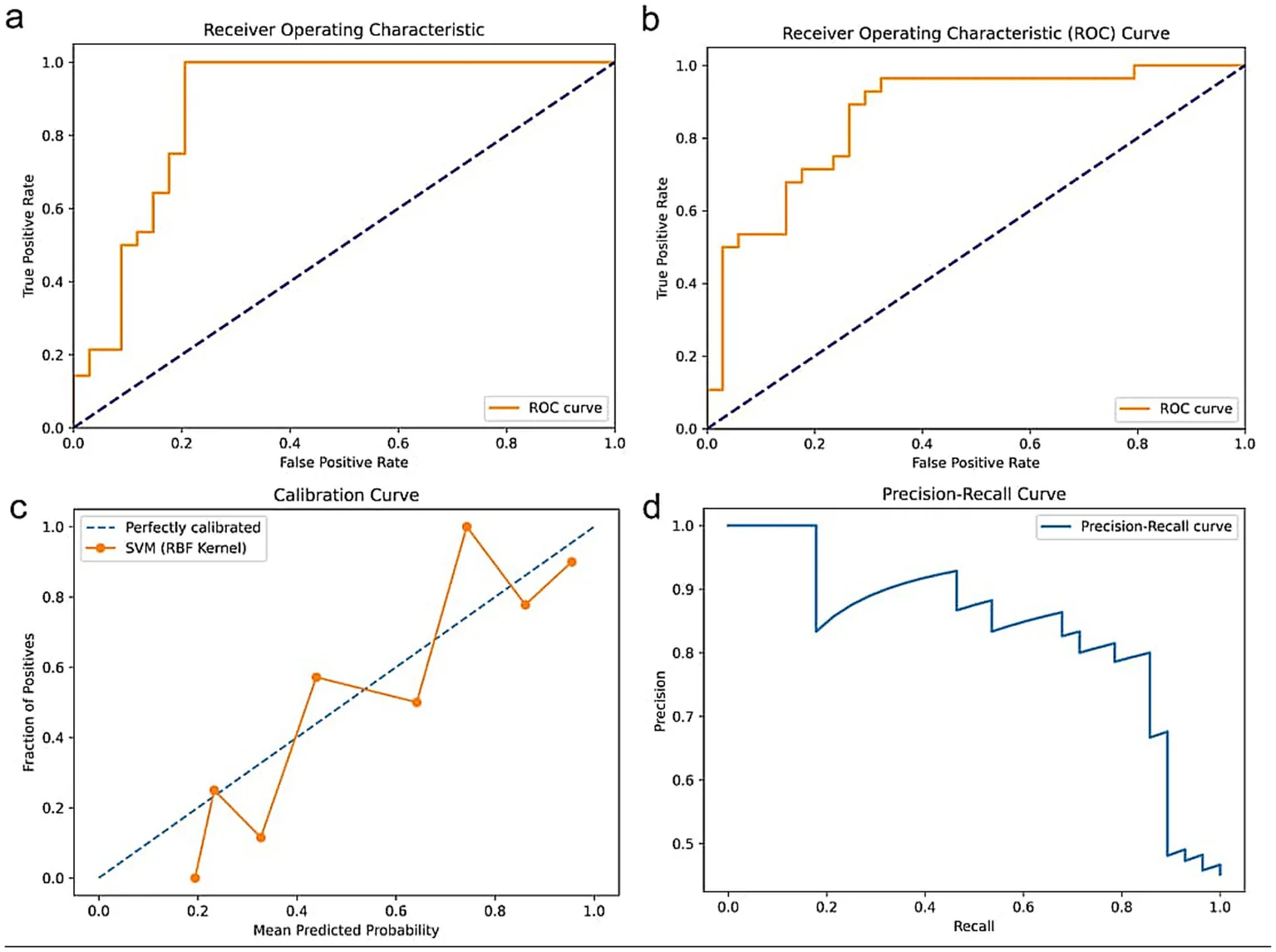
Performance evaluation of the SVM model. **(a)** Receiver operating characteristic (ROC) curve of the SVM model evaluated within the development cohort using nested cross-validation after inclusion of five features. **(b)** ROC curve of the SVM model in the independent external validation cohort after inclusion of five features. **(c)** Calibration curve of the SVM model in the external validation cohort. **(d)** Precision–Recall (P–R) curve of the SVM model in the external validation cohort.

The changes in predictive performance of the SVM model with features added sequentially according to SHAP-based importance ranking are summarized in Table 4. When 5 features were included, the SVM model achieved its optimal discriminative performance during internal validation based on nested cross-validation, with a mean AUROC of 0.873, sensitivity of 0.784, and specificity of 0.897 (Figure 5a). Based on these results, 5 variables were selected for inclusion in the final SVM model. To further evaluate the apparent performance difference, we estimated the uncertainty of the CRP-only model using bootstrap resampling (2,000 iterations) within the development cohort. The AUROC of the CRP-only model was 0.889 (95% CI: 0.852–0.921), which overlapped substantially with that of the multivariable model (AUROC: 0.873, 95% CI: 0.842–0.904). To evaluate the impact of Borderline-SMOTE, we additionally trained the SVM model without oversampling but with class weights inversely proportional to class frequencies. The model without SMOTE achieved a mean AUROC of 0.862 (95% CI: 0.83–0.89), sensitivity 0.76, and specificity 0.88 in internal validation; performance was comparable to that with SMOTE (AUROC: 0.870). In the external cohort, the non-SMOTE model yielded an AUROC of 0.855 (95% CI: 0.82–0.88). This suggests that the model’s discrimination is not highly sensitive to the resampling strategy, and the use of Borderline-SMOTE may be safely omitted in future applications, particularly given the modest degree of class imbalance.

**Table 4:** The prediction performance changes of model with different variables.

Number of features	AUROC (95% CI)	Sensitivity	Specificity	Variables in model
1	0.889 (0.852–0.921)	0.882	0.750	“CRP”
2	0.860 (0.831–0.889)	0.893	0.824	“CRP, NPS”
3	0.862 (0.833–0.891)	0.892	0.825	“CRP, NPS, PLR”
4	0.830 (0.800–0.860)	0.824	0.779	“CRP, NPS, PLR, SII”
5	0.873 (0.842–0.904)	0.784	0.897	“CRP, NPS, PLR, SII, GPS”

Model performance was further evaluated in an independent validation cohort. In this cohort, the model achieved an AUROC of 0.862, with a sensitivity of 0.750 and a specificity of 0.824 (Figure 5b). The calibration curve demonstrated reasonable agreement between predicted and observed probabilities (Figure 5c), with a calibration slope of 1.142 and an intercept of −0.113. The calibration-in-the-large estimate (mean predicted probability minus observed event rate) was 0.09, indicating a systematic overestimation of risk by approximately 9% in the external cohort. As an exploratory supplementary analysis, a *post hoc* logistic recalibration analysis was conducted in the external cohort to assess whether probability estimates could be further improved. After recalibration, the calibration slope improved to 1.03 and the intercept to −0.02, indicating better agreement between predicted and observed probabilities. The Brier score showed a slight improvement after recalibration (from 0.102 to 0.098). However, because this recalibration was performed on the same external cohort, the improvement should be interpreted as optimistic and not as evidence of generalizable calibration performance.

**Figure 6:**
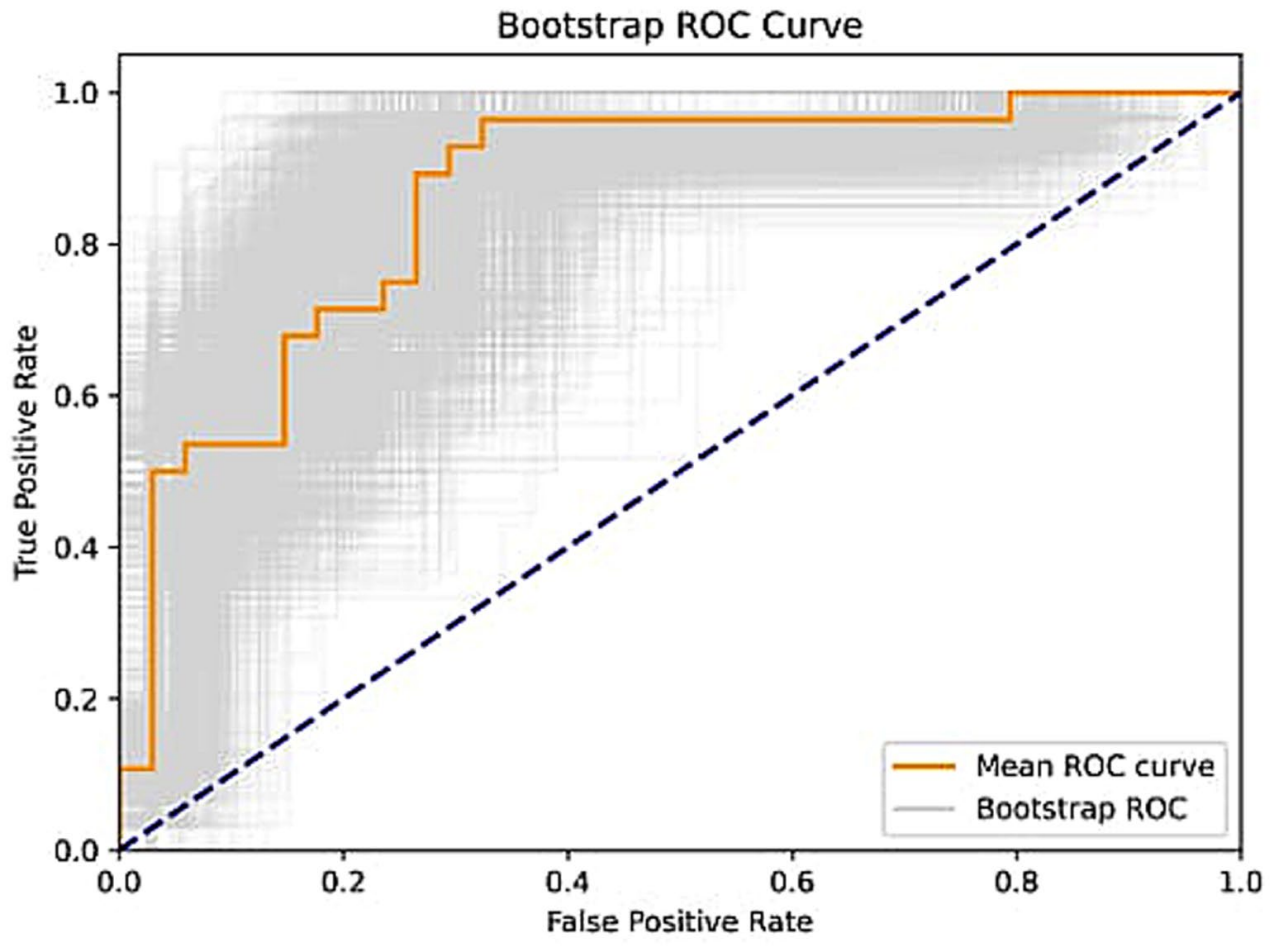
Bootstrap validation of model performance in the external cohort. Model performance was assessed in the external validation cohort (*n* = 152) from the First Affiliated Hospital of Shanxi Medical University. Bootstrap resampling (2,000 replications) was applied to the external cohort to estimate 95% confidence intervals for performance metrics.

Precision–recall analysis showed an average precision of 0.841 across different recall thresholds, indicating stable performance under class imbalance (Figure 5d). To quantify uncertainty in model performance, bootstrap resampling (2,000 iterations) was applied to the external validation cohort of 152 patients, yielding an AUROC of 0.862(95% CI: 0.829–0.884), with corresponding sensitivity and specificity of 0.741 and 0.841, respectively (Figure 6). The comprehensive performance of the model across both development and validation phases is consolidated in Table 5. Internally, the 5-fold nested cross-validation on the retrospective cohort (*n* = 350) provided an unbiased performance estimate, yielding an AUROC of 0.870. The optimal operational threshold was determined as 0.42 by maximizing Youden’s index during this phase. To rigorously assess generalizability, the final model—with its fixed configuration and this predetermined threshold—was applied to the independent prospective cohort (*n* = 152). The model maintained good discrimination (AUROC: 0.862) in this external setting. The broadly comparable performance between internal and external validation supports the potential usefulness of the model for risk stratification, although further prospective evaluation remains necessary.

**Table 5:** Model performance in internal and external validation.

Performance metric	Internal validation (5 - fold Nested CV, *n* = 350)		External validation (prospective cohort, *n* = 152)	
	Value	95% CI	Value	95% CI
Discrimination and calibration				
Area under the ROC curve (AUROC)	0.870	0.840–0.900	0.862	0.829–0.884
Average precision (AP)	0.841	0.80–0.88	0.835	0.79–0.87
Brier score	0.105	0.09–0.12	0.102	0.085–0.119
Calibration slope	1.00	0.88–1.12	1.142	0.95–1.33
Calibration intercept	0.00	−0.10–0.10	−0.113	−0.25–0.02
Classification metrics at threshold = 0.42				
Sensitivity (recall)	0.784	0.72–0.84	0.750	0.65–0.83
Specificity	0.897	0.86–0.93	0.824	0.76–0.88
Positive predictive value (precision)	0.812	0.75–0.87	0.714	0.62–0.80
Negative predictive value	0.875	0.83–0.91	0.852	0.79–0.90
Accuracy	0.857	0.82–0.89	0.796	0.73–0.85
F1 - score	0.798	0.74–0.85	0.731	0.65–0.80

Internal validation results were derived from 5-fold nested cross-validation on the development cohort (*n* = 350). External validation was performed on the independent prospective cohort (*n* = 152). The operational threshold of 0.42 was determined by maximizing Youden’s index during internal validation. Confidence intervals for internal validation were derived from nested cross-validation combined with bootstrap resampling.

To formally compare the prognostic value of the five-variable model versus CRP alone, we performed DeLong’s test for paired ROC curves and calculated the net reclassification improvement (NRI) and integrated discrimination improvement (IDI). The DeLong test showed that the AUROC difference between the two models was not statistically significant (difference = 0.016, 95% CI: −0.031 to 0.063, *p* = 0.182). The NRI was 4.8% (95% CI: −2.3 to 11.9%) and the IDI was 0.009 (95% CI: −0.006 to 0.024), indicating only marginal incremental value beyond CRP alone. Decision curve analysis (Supplementary Figure S2) demonstrated that the multivariable model provided a modest net benefit over the ‘treat-all’ strategy across a threshold range of 0.2–0.5, but was largely comparable to the CRP-only model. Although the multivariable model did not significantly improve overall discrimination beyond CRP alone, it provides complementary inflammatory information and may facilitate a more comprehensive assessment of inflammatory status during clinical risk stratification.

Although the AUROC showed a non-monotonic pattern (dropping from 0.862 to 0.830 when adding SII, then rising to 0.873 with GPS), the bootstrap 95% confidence intervals for adjacent steps overlapped substantially (e.g., 3-feature CI: 0.833–0.891 vs. 4-feature CI: 0.800–0.860), suggesting that the variation is within sampling error rather than reflecting genuine model instability.

In the internal nested cross-validation, the mean AUROC (95% CI) for penalized logistic regression was 0.861 (0.83–0.89), for random forest 0.855 (0.82–0.88), and for XGBoost 0.863 (0.83–0.89), compared to 0.870 (0.840–0.900) for SVM. The Brier scores were 0.112, 0.118, and 0.109, respectively, versus 0.105 for SVM. SVM was selected as the final model due to its marginally superior discriminative performance and favorable calibration. All comparison models were trained using the identical nested cross-validation framework, feature selection procedure, preprocessing pipeline, and hyperparameter optimization strategy.

### Model fairness analysis

To evaluate potential performance bias, a fairness analysis was conducted by examining the interaction between the model’s predicted probability (PP) and key clinical characteristics (Table 6). Logistic regression analysis showed that all interaction terms—specifically Age × PP, Sex × PP, and Treatment interventions × PP—were not statistically significant (all *p* > 0.05). This indicates that the SVM model’s predictive performance remains consistent and unbiased across different demographic groups and clinical treatment protocols. Interestingly, the main effect of sex was statistically significant, suggesting potential biological or healthcare-related differences that warrant further investigation in future studies.

**Table 6:** Fairness analysis: associations between predicted probability and observed outcomes across age, sex, and treatment subgroups.

Variable	Coefficient (β)	Standard error	*p*-value
Intercept	−0.1576	0.0325	< 0.001
Age	0.0004	0.0006	0.482
Sex	0.0289	0.0141	0.042
Decompressive craniectomy	0.0066	0.0181	0.715
Thrombectomy	0.0551	0.0204	0.285
Anticoagulation therapy	0.0036	0.0064	0.582
Age×PP	−0.0015	0.0012	0.223
Sex×PP	−0.0018	0.0023	0.420
Decompressive craniectomy×PP	0.0421	0.0307	0.173
Thrombectomy×PP	0.0761	0.0573	0.320
Anticoagulation therapy×PP	0.0549	0.0368	0.264
Predicted probability(PP)	1.3093	0.0564	4.63E-48

The model’s robust generalizability was further corroborated through exploratory subgroup analyses within the external validation cohort. Model discrimination and error rates were evaluated using the area under the receiver operating characteristic curve (AUROC), true positive rate (TPR), and false positive rate (FPR), with uncertainty quantified via bootstrap-derived 95% confidence intervals (CI).

In sex-stratified analyses, the SVM model demonstrated comparable discriminative performance between groups, with an AUROC of 0.86 (95% CI: 0.81–0.90) for male patients and 0.85 (95% CI: 0.79–0.90) for female patients. Sensitivity and false positive rates were also similar across groups (Table 7), indicating no substantial performance disparity attributable to sex. Calibration parameters were also assessed separately by sex. In male patients, the calibration slope was 1.10 (95% CI: 0.92–1.28) and intercept −0.09 (95% CI: −0.22 to 0.04); in female patients, the calibration slope was 1.18 (95% CI: 0.96–1.40) and intercept −0.12 (95% CI: −0.28 to 0.04). Although the confidence intervals overlapped substantially, slightly poorer calibration was observed in females, which may reflect the smaller sample size in this subgroup.

**Table 7:** Sex-based subgroup performance in the external validation cohort.

Sex	AUROC (95% CI)	Sensitivity/TPR (95% CI)	FPR (95% CI)
Male	0.86 (0.81–0.90)	0.75 (0.68–0.82)	0.17 (0.12–0.22)
Female	0.85 (0.79–0.90)	0.73 (0.65–0.81)	0.18 (0.13–0.24)
(Male − Female)	0.01	0.02	−0.01

Age-stratified analyses yielded consistent results. For patients younger than 50 years, the model achieved an AUROC of 0.87 (95% CI: 0.82–0.91), while for those aged 50 years or older, the AUROC was 0.84 (95% CI: 0.78–0.89) (Table 8). Although the older subgroup showed slightly lower sensitivity and higher false positive rates, the substantial overlap in confidence intervals suggests no strong evidence of age-related performance inequity. Overall, these findings support the stability and fairness of the proposed model across diverse demographic and clinical strata.

**Table 8:** Age-based subgroup performance in the external validation cohort.

Age group	AUROC (95% CI)	Sensitivity/TPR (95% CI)	FPR (95% CI)
< 50 years	0.87 (0.82–0.91)	0.76 (0.69–0.83)	0.16 (0.11–0.21)
≥ 50 years	0.84 (0.78–0.89)	0.72 (0.64–0.80)	0.19 (0.14–0.25)
(<50 − ≥50)	0.03	0.04	−0.03

AUROC, sensitivity (TPR), and false positive rate (FPR) were calculated in the external validation cohort. Confidence intervals were estimated using bootstrap resampling (2,000 iterations). Subgroup analyses were predefined and exploratory.

## Discussion

Inflammation is recognized as a significant factor in arterial stroke, contributing to its onset, progression, and influencing outcomes (26–28). Similarly, numerous studies have identified inflammation acting as the cause of CVT and explored its correlation with CVT prognosis (4, 11, 29, 30). However, predictive models in this area remain scarce. In this study, we presented a CVT prognosis prediction model using SVM algorithm. Our research has further established and validated a machine learning based long-term CVT prognosis prediction model, underscoring its potential clinical relevance. This study pioneers the establishment of a multicenter framework that integrates prospective and retrospective methodologies to investigate inflammation’s role in CVT, while introducing GPS and NPS for the first time to evaluate their association with CVT prognosis.

Over the past few years, intracranial vascular research has shifted its focus from arteries to veins, particularly on conditions such as CVT. With an incidence of 1.3 to 1.6 per 100,000 (2, 31), CVT, a less common stroke subtype, has seen improved detection rates due to advancements in imaging (5). In this retrospective multicenter analysis of 350 patients, we incorporated routinely available clinical and laboratory variables. Using the SVM algorithm, we developed a prediction model based on clinical parameters and laboratory tests. In internal validation using nested cross-validation, the model achieved an AUROC of 0.870, with a sensitivity of 0.784 and a specificity of 0.897. In the independent external validation cohort, the model maintained excellent discrimination, achieving an AUROC of 0.862, with sensitivity and specificity of 0.750 and 0.824, respectively.

In the realm of data processing, handling missing values and addressing class imbalance are critical challenges in clinical machine learning. Missing data were assumed to be missing at random based on clinical review, and missing-indicator variables were introduced to capture potential information carried by missingness patterns, in combination with mean or mode imputation for continuous and categorical variables, respectively (32). The robustness of the imputation strategy was further supported by sensitivity analysis using multiple imputation, which showed consistent model performance and feature selection results.

Although the imbalance between positive and negative outcomes in the development cohort was moderate, adverse outcomes are of particular clinical importance in CVT prognosis prediction (33). To reduce potential bias toward the majority class while limiting the risk of overfitting, we adopted the Borderline-SMOTE approach (25), which focuses oversampling on samples near the decision boundary and has been shown to improve discrimination in imbalanced medical datasets compared with conventional SMOTE methods (34). Over-sampling (Borderline-SMOTE) was applied only within the inner-loop training folds; no resampling was performed in outer validation folds or in the external cohort. In high-dimensional regression, the judicious selection of features is important. While LASSO is renowned for its feature selection capabilities, it can falter in the presence of multicollinearity (35). Elastic Net (Enet), blending LASSO and Ridge regression, addresses this by ensuring both sparsity and stability (36).

In our study, with 31 initial features, we first employed LASSO, addressing convergence through iterative refinement. Recognizing potential multicollinearity challenges, we innovatively integrated cv-Enet for feature selection. This harmonization yielded 11 salient variables. In our prognostic model, CRP, NPS, PLR, SII, and GPS were the five final indicators selected (Table 4). All five markers are readily obtainable from routine blood tests in clinical settings. Our analysis of predictive performance changes demonstrated that the inclusion of these five variables optimized the balance between sensitivity and specificity. Notably, while CRP as a standalone marker demonstrates high sensitivity, its specificity is relatively limited; the addition of composite inflammatory-coagulative indices like NPS and GPS contributed to improved specificity and a more balanced classification profile.

CRP is a well-established acute-phase reactant and has been consistently associated with thrombosis and vascular inflammation (33). In recent years, the role of CRP in CVT has garnered significant attention (29, 37–39). Gogu et al. (40) found a correlation between elevated CRP levels and the exacerbation of CVT. Concurrently, Tekesin et al. (41) proposed that CRP, combined with other inflammatory markers, can enhance the diagnostic and prognostic assessment. These findings align with our observations. When solely using CRP as a predictive marker, it achieved an AUROC of 0.889, with a high sensitivity of 0.882, but a specificity of only 0.75. This suggests that while CRP demonstrates high sensitivity (42), its value as a standalone predictor is limited. For enhanced predictive accuracy, CRP should be combined with other biomarkers. Although CRP alone achieved a slightly higher AUROC, this difference is likely attributable to sampling variability and the relatively limited sample size. In this cohort, CRP may capture a substantial proportion of prognostic information, while the addition of correlated inflammatory markers may introduce redundancy and noise, slightly reducing apparent discrimination. Importantly, the multivariable model demonstrated a more balanced trade-off between sensitivity and specificity, with substantially improved specificity (0.897 vs. 0.750), which is clinically valuable for reducing false-positive predictions. Therefore, although the multivariable model did not significantly improve discrimination over CRP alone, its higher specificity and multi-marker profile may provide complementary information for clinical risk stratification.

The comparable performance of the CRP-only and multivariable models suggests that the incremental discriminatory value of additional inflammatory markers beyond CRP may be limited in this cohort. CRP alone showed strong discriminative ability, indicating that it captured a substantial proportion of the prognostic signal. However, the multivariable model provided a more balanced sensitivity–specificity profile and incorporated complementary inflammatory indices, providing additional information that may support clinical risk stratification when used alongside CRP.

PLR reflects the balance between inflammatory and coagulative pathways (43–46). An elevated PLR was associated with increased adverse outcomes in our cohort, with a SHAP value of 1.055 (Figure 4b), consistent with previous findings linking higher PLR to worse prognosis in thrombotic conditions.

SII integrates platelet, neutrophil, and lymphocyte counts and reflects the interplay between inflammatory and immune pathways (46). Platelets contribute to hemostasis, inflammation, and thrombosis (47), while neutrophils and lymphocytes mediate innate and adaptive immunity, respectively (48, 49). Elevated SII may therefore indicate enhanced inflammation and immune dysregulation, increasing thrombotic risk. Emerging evidence supports SII as a prognostic marker in acute/subacute CVST (50, 51), consistent with our finding that higher SII values were associated with poor outcomes in this cohort.

In a study involving 270 patients (52), a significant association was found between high SII values and adverse outcomes in CVST, particularly an increased mortality rate. This aligns with our findings, further emphasizing the significance of SII in CVT, especially its correlation with unfavorable outcomes.

Inflammatory and immunological processes are central to the pathogenesis and clinical outcomes of numerous disorders. Over time, as our comprehension of inflammation’s role in disease has deepened, several inflammatory indices have been introduced and integrated into clinical settings (15, 16, 53). GPS and NPS are prominent among them. The GPS evaluates systemic inflammatory responses based on CRP and albumin levels (15, 54, 55), while the NPS reflects the balance of inflammation and coagulation by combining neutrophil and platelet counts (56). Although these indices have been linked to adverse outcomes in other conditions, their significance in CVT remains underexplored. The mechanisms of action of GPS and NPS in other diseases might be analogous to their potential mechanisms in CVT. They may reflect systemic inflammatory responses and the balance of coagulation, thereby influencing disease progression and prognosis (54–56). In our study, we may be the first to reveal the association between NPS, GPS, and the prognosis of CVT. This discovery not only offers a fresh perspective for the prognostic evaluation but also further confirms the central role of inflammation in CVT.

NLR and LMR have garnered significant attention in inflammation research over the past few years. Studies have shown that, compared to patients with chronic CVT, those with acute CVT exhibit significantly elevated NLR levels (29, 52, 57). Moreover, NLR correlates positively with the baseline disability degree in CVT patients, making it a vital tool for predicting the prognosis of severe CVT patients. A plethora of evidence supports the notion that an increased NLR level is significantly associated with adverse outcomes in CVT patients (44, 57). Thus, elevated NLR levels upon admission might indicate a heightened risk of long-term adverse outcomes for these patients (29, 39, 51).

On the other hand, the LMR, which reflects the balance between lymphocytes and monocytes, provides crucial insights into a patient’s baseline inflammatory and immune status. In stroke patients, variations in lymphocyte and monocyte levels are closely related to prognosis (58). Notably, LMR has a negative correlation with the severity of stroke. Some contemporary retrospective studies further suggest that a low LMR level upon admission might be associated with adverse outcomes in severe CVT patients (59). As the disease is treated and recedes, we have also observed a gradual rise in LMR levels.

In this study, we selected SVM as the final predictive algorithm (19). Prognostic prediction in CVT may involve nonlinear relationships among inflammatory biomarkers and clinical variables, and the kernel function of SVM can help capture such nonlinear patterns (60). Given the limited sample size and the moderate dimensionality of the dataset, SVM provides a relatively established modeling framework that may reduce the risk of overfitting compared with more complex algorithms (17, 18). After isotonic calibration (61), the calibration curve showed partial agreement between predicted and observed probabilities, with a slope of 1.142 and an intercept of −0.113. These findings suggest that predicted probabilities may be useful for risk stratification, but they should be interpreted cautiously when used for absolute risk estimation in new populations.

Although the external validation demonstrated good discriminative performance, calibration remained imperfect, with a calibration slope greater than 1 suggesting a tendency toward overconfident predictions in the external cohort. As an exploratory supplementary analysis, *post hoc* logistic recalibration improved both the calibration slope and intercept, indicating better agreement between predicted and observed risks. Previous studies have emphasized that recalibration may improve probability estimation when prediction models are applied to new populations (62). Therefore, the current model may be more appropriately interpreted as a risk stratification tool, and recalibration may be beneficial before direct probability estimation in different clinical settings.

Precision–recall analysis was used as a complementary assessment because poor outcomes were less frequent than good outcomes in the development cohort. The model achieved an average precision of 0.841, supporting its discriminative ability under moderate class imbalance. Nevertheless, this result should be interpreted as supportive rather than definitive evidence of clinical utility, and further prospective evaluation is needed before routine clinical implementation.

We further assessed the consistency of model performance across key demographic and clinical subgroups by examining potential interaction effects between predicted probabilities and variables including age, sex, and therapeutic interventions. Fairness assessment has become an increasingly important consideration in clinical machine learning research (63). No strong evidence of differential associations was observed across these subgroups, suggesting that model performance was broadly consistent in clinically relevant populations.

The absence of a significant interaction with surgical intervention may reflect the clinical characteristics of the study population, in which surgical procedures were primarily performed in patients with more severe disease at presentation. In this context, surgical intervention may function as a marker of disease severity rather than an independent modifier of long-term prognosis. Therefore, the lack of a detectable interaction effect should not be interpreted as evidence of limited clinical benefit of surgery, but rather as a reflection of case-mix heterogeneity and limited statistical power.

Overall, these exploratory analyses did not reveal strong evidence of systematic performance disparities across age, sex, or treatment subgroups. Given the modest sample size and the exploratory nature of these analyses, the results should be interpreted cautiously, and further validation in larger and more diverse cohorts is warranted.

In essence, we developed an accurate and robust CVT prognosis prediction model. Exploratory interaction and subgroup analyses did not reveal strong evidence of systematic performance differences across sex, age, or surgical intervention subgroups. These findings suggest that the model demonstrates broadly consistent performance across key demographic and clinical strata. Nevertheless, given the limited sample size and exploratory nature of these analyses, further validation in larger and more diverse cohorts is warranted to confirm the fairness and generalizability of the proposed model.

This study has several limitations. The 62% male predominance in our cohort is atypical for CVT, which generally affects women more frequently. This discrepancy may reflect referral patterns, the exclusion of pregnancy- and oral contraceptive-related CVT, or regional demographic differences. It warrants consideration when applying the model to populations with a higher female proportion. Our retrospective design inherently introduced selection bias and missing data, which could impede the conclusiveness typically achieved in prospective trials with systematic patient recruitment and data collection. Nevertheless, we adhered to the TRIPOD guidelines (64), ensuring rigorous reporting. Although an independent prospective external validation cohort was available, its sample size was relatively modest, which may limit the precision of subgroup and fairness analyses. Furthermore, the limited number of outcome events (*n* = 112) relative to the 31 candidate predictors yielded a low events-per-variable ratio (3.6), which is well below the recommended minimum of 10–20. Despite the use of nested cross-validation and stringent feature selection, the risk of overfitting remains elevated, and the estimates of model performance may be optimistic. Larger cohorts are needed to achieve adequate EPV and to enable robust external calibration. To further quantify uncertainty, we additionally applied bootstrap resampling in the external cohort. Larger multicenter prospective studies are warranted to further confirm the generalizability of the proposed model. We also faced data imbalance, a common challenge in medical research (65). To address this, we utilized the Borderline SMOTE oversampling technique (25), effectively balancing the data. While larger datasets often optimize machine learning models (17, 18), our model demonstrated notable accuracy given the limited events in our dataset. Future large-scale multicenter prospective studies will be essential to further validate the efficacy of our ML model.

## Conclusion

This study developed and externally validated an SVM-based prognostic model using five inflammatory markers for 3-month functional outcome in CVT. The model’s discriminative performance was comparable to that of CRP alone on AUROC, but it provided higher specificity and retained a multi-marker inflammatory profile. Given the modest sample size, low events-per-variable ratio, and residual calibration drift, the model should be considered an exploratory adjunct for risk stratification rather than a definitive clinical tool. Further large-scale multicenter prospective studies are required to confirm its incremental value and generalizability.

## Data Availability

The raw data supporting the conclusions of this article will be made available by the authors, without undue reservation.

## Glossary

AIC: Akaike information criterion
AIS: Acute ischemic stroke
AP: Average precision
ANOVA: analysis of variance
AUC: Area under the curve
BIC: Bayesian information criterion
Borderline-SMOTE: Borderline synthetic minority oversampling technique
CI: Confidence interval
CRP: C-reactive protein
CRP/Alb ratio: C-reactive protein-to-albumin ratio
CVT: Cerebral venous thrombosis
DVT: Deep vein thrombosis
Enet: Elastic Net
GPS: Glasgow Prognostic Score
LASSO: Least absolute shrinkage and selection operator
LMR: Lymphocyte-to-monocyte ratio
ML: Machine learning
MRI: Magnetic resonance imaging
MRV: Magnetic resonance venography
mRS: modified Rankin Scale
NLR: Neutrophil-to-lymphocyte ratio
NPS: Neutrophil–platelet score
NPV: Negative predictive value
PE: Pulmonary embolism
PLR: Platelet-to-lymphocyte ratio
PP: Predicted probability
PPV: Positive predictive value
PR: Precision–recall
RF: Random forest
ROC: Receiver operating characteristic
SHAP: SHapley Additive exPlanations
SII: Systemic immune-inflammation index
SVM: Support vector machine
TRIPOD: Transparent Reporting of a multivariable prediction model for Individual Prognosis or Diagnosis
XGBoost: eXtreme Gradient Boosting
