# Supplementary material for: Predicting 3-month prognosis of cerebral venous thrombosis: a machine learning approach incorporating inflammatory markers
Source: Front Neurol. 2026 Aug 11;17:1843121. doi: 10.3389/fneur.2026.1843121 (PMC13503163; doi:10.3389/fneur.2026.1843121)
Supplement: Supplementary file 1 [file Supplementary_file_1.DOCX]

**Supplementary Methods S1**

Input:

Dataset D with features X and outcome Y

Outer folds K = 5

Inner folds J = 5

Random seed = 42

Output:

Final trained model and performance estimates

Procedure:

1. Initialize random seed for reproducibility

2. Split dataset D into K outer folds:

For each outer fold k = 1 to K:

2.1 Define:

Training set D_train_outer

Test set D_test_outer

2.2 Within D_train_outer, perform J-fold inner cross-validation:

For each inner fold j = 1 to J:

2.2.1 Split:

D_train_inner

D_valid_inner

2.2.2 Preprocessing (fit only on D_train_inner):

	-Impute missing values using parameters estimated from D_train_inner only

-Standardize features using parameters estimated from D_train_inner only

-Apply the fitted preprocessing parameters to D_valid_inner without refitting

2.2.3 Class-imbalance handling:

	-Apply Borderline-SMOTE only to the preprocessed D_train_inner

	-Do not apply oversampling to D_valid_inner, D_test_outer, or the external validation cohort

2.2.4 Feature selection (performed only on the resampled D_train_inner):

	-Apply LASSO regression with cross-validated lambda

	-Apply Elastic Net regression with cross-validated alpha and lambda

	-Take the intersection of features selected by LASSO and Elastic Net

2.2.5 Feature refinement:

- Train Random Forest on selected features

- Rank features by importance

- Retain top N features based on importance threshold

2.2.6 Model training:

- Train SVM with a radial basis function (RBF) kernel using the selected features

- Tune hyperparameters via grid search:

C ∈ {0.01, 0.1, 1, 10, 100}

γ ∈ {0.001, 0.01, 0.1, 1}

2.2.7 Validation:

- Evaluate model on D_valid_inner

- Record AUROC

2.3 Select the optimal hyperparameters based on mean inner-fold AUROC

2.4 Retrain model on full D_train_outer:

- Repeat preprocessing and feature selection steps using entire D_train_outer

- Train final SVM with selected hyperparameters

2.5 Evaluate on D_test_outer:

- Compute AUROC, sensitivity, specificity

3. Aggregate performance across K outer folds:

- Report mean AUROC and 95% confidence intervals

4. Train final model on full development dataset using optimal parameters

5. External validation:

- Apply trained model to independent cohort

- Evaluate discrimination and calibration

**Supplementary Table S1. Proportion of Missing Data for Each Variable in the Development Cohort (n = 350)**

| **Variable** | **Development cohort** $\text{n=350}$ | **External validation cohort** $\text{n=152}$ | **Overall** $\text{n=502}$ |
| --- | --- | --- | --- |
| Age | 3 (0.9%) | 1 (0.7%) | 4 (0.8%) |
| Sex | 0 (0.0%) | 0 (0.0%) | 0 (0.0%) |
| Hypertension | 5 (1.4%) | 2 (1.3%) | 7 (1.4%) |
| Diabetes | 4 (1.1%) | 0 (0.0%) | 4 (0.8%) |
| Smoking | 6 (1.7%) | 3 (2.0%) | 9 (1.8%) |
| Headache | 2 (0.6%) | 1 (0.7%) | 3 (0.6%) |
| Consciousness dysfunction | 4 (1.1%) | 2 (1.3%) | 6 (1.2%) |
| Heart rate | 3 (0.9%) | 4 (2.6%) | 7 (1.4%) |
| Respiratory rate | 3 (0.9%) | 1 (0.7%) | 4 (0.8%) |
| White blood cell | 7 (2.0%) | 2 (1.3%) | 9 (1.8%) |
| Hemoglobin | 6 (1.7%) | 3 (2.0%) | 9 (1.8%) |
| C-reactive protein (CRP) | 8 (2.3%) | 3 (2.0%) | 11 (2.2%) |
| Platelet | 7 (2.0%) | 1 (0.7%) | 8 (1.6%) |
| Thrombin time | 10 (2.9%) | 4 (2.6%) | 14 (2.8%) |
| Fibrinogen | 9 (2.6%) | 2 (1.3%) | 11 (2.2%) |
| Prothrombin time | 11 (3.1%) | 3 (2.0%) | 14 (2.8%) |
| Lactate | 12 (3.4%) | 4 (2.6%) | 16 (3.2%) |
| Lactate dehydrogenase | 10 (2.9%) | 3 (2.0%) | 13 (2.6%) |
| AST | 11 (3.1%) | 2 (1.3%) | 13 (2.6%) |
| ALT | 9 (2.6%) | 1 (0.7%) | 10 (2.0%) |
| LMR | 13 (3.7%) | 3 (2.0%) | 16 (3.2%) |
| NLR | 14 (4.0%) | 4 (2.6%) | 18 (3.6%) |
| PLR | 12 (3.4%) | 2 (1.3%) | 14 (2.8%) |
| SII | 13 (3.7%) | 3 (2.0%) | 16 (3.2%) |
| GPS | 6 (1.7%) | 1 (0.7%) | 7 (1.4%) |
| NPS | 7 (2.0%) | 2 (1.3%) | 9 (1.8%) |

**Supplementary Table S2. Pearson correlation matrix among the five final predictors in the development cohort (n = 350).**

| Variable | CRP | PLR | SII | NPS | GPS |
| --- | --- | --- | --- | --- | --- |
| CRP | 1.00 |  |  |  |  |
| PLR | 0.38 | 1.00 |  |  |  |
| SII | 0.41 | 0.62 | 1.00 |  |  |
| NPS | 0.29 | 0.35 | 0.44 | 1.00 |  |
| GPS | 0.55 | 0.32 | 0.40 | 0.37 | 1.00 |

Values are Pearson correlation coefficients (r). All correlations were statistically significant (p < 0.001). CRP, C‑reactive protein; PLR, platelet‑to‑lymphocyte ratio; SII, systemic immune‑inflammation index; NPS, neutrophil‑platelet score; GPS, Glasgow Prognostic Score.

**Supplementary FigureS1. Patient enrollment flowchart.**

Flowchart showing patient selection for the development (n=350) and external validation (n=152) cohorts. *Some patients met multiple exclusion criteria.

**Supplementary FigureS2. Decision curve analysis for the SVM model in the external validation cohort.**

Net benefit of the five‑variable SVM model, CRP‑alone model, “treat all,” and “treat none” strategies across threshold probabilities. The vertical dashed line marks the operating threshold of 0.42.
